# Bibliography

**Published:** 1898-03

**Authors:** 


### Bibliography.

THE INDIANA DENTAL JOURNAL. Edited by George Edwin Hunt, M.D., D.D.S. Vol. I., No. 1. Indianapolis, January, 1898.

Another candidate for professional favor has been sent forth to the world from Indianapolis, with Dr. Hunt as editor.

The first number presents a neat appearance and is typographically very satisfactory. It is to be published monthly by the "Indiana Dental Journal Company." Exactly what that may mean is not stated, but it would be a gratification if it were proved to be entirely independent of supply houses.

In wishing it entire success, it is to be regretted that it is started on lines which, if continued, will not redound to the honor or dignity of the dental profession. Dentistry has had much to contend with in the past, and has yet a large number connected with it who fail to see that a dignified literature is as equally important as dignified personal bearing, if the respect of the intelligent, thinking world is desired. Slang, cheap wit, rhymes, and novelettes are not exactly the mental food to spread before professional men at the close of the nineteenth century. It is most desirable that the editor should see his way out of this literary mire and eventually give the twentieth century a journal worthy of his name and reputation.

A Treatise on Irregularities of the Teeth and their Correction. By John Nutting Farrar, M.D., D.D.S., Esq. The International News Company: New York, London, Leipsic, 1888–97.

The second volume of this great work of Dr. Farrar came to us too late for extended notice in this number, a full review being necessarily deferred until the April issue. A cursory examination of the contents leads to the conclusion that this volume more than sustains the reputation of the author, treating the practical side of irregularities of the teeth so exhaustively, and at the same time with such clearness and simplicity of expression, that all who read cannot fail to learn.

This volume has been prepared with the same care as that given to the first, both of them being superior specimens of the printing art.

